# Muscle mass, muscle strength and mortality in kidney transplant recipients: results of the TransplantLines Biobank and Cohort Study

**DOI:** 10.1002/jcsm.13070

**Published:** 2022-10-06

**Authors:** Iris M.Y. van Vliet, Adrian Post, Daan Kremer, Karin Boslooper‐Meulenbelt, Yvonne van der Veen, Margriet F.C. de Jong, Robert A. Pol, TransplantLines Investigators, Harriët Jager‐Wittenaar, Gerjan J. Navis, Stephan J.L. Bakker, G. Dijkstra, V.E. de Meijer, H.G.D. Leuvenink, S.J.L. Bakker, C.T. Gan, J.S.F. Sanders, E.A.M. Verschuuren, K. Damman, W.S. Lexmond, J. Blokzijl, M.H. de Borst, M.E. Erasmus, R.J. Porte, M.T. de Boer, R.A. Pol, S.P. Berger, M.F. Eisenga, A.W. Gomes Neto, D. Kremer, M. van Londen, J.H. Annema‐de Jong, M.J. Siebelink, L.J. van Pelt, H.G.M. Niesters, F.A.J.A. Bodewes, B.G. Hepkema, A.V. Ranchor, R.M. Douwes

**Affiliations:** ^1^ Department of Dietetics University of Groningen, University Medical Center Groningen Groningen The Netherlands; ^2^ Department of Internal Medicine, Division of Nephrology University of Groningen, University Medical Center Groningen Groningen The Netherlands; ^3^ Department of Surgery University of Groningen, University Medical Center Groningen Groningen The Netherlands; ^4^ Groningen Transplant Center University of Groningen, University Medical Center Groningen Groningen The Netherlands; ^5^ Department of Oral and Maxillofacial Surgery University of Groningen, University Medical Center Groningen Groningen The Netherlands; ^6^ Research Group Healthy Ageing, Health Care and Nursing Hanze University of Applied Sciences Groningen The Netherlands

**Keywords:** bio‐electrical impedance analysis, hand grip strength, kidney transplant, mortality, muscle mass, muscle strength

## Abstract

**Background:**

Survival of kidney transplant recipients (KTR) is low compared with the general population. Low muscle mass and muscle strength may contribute to lower survival, but practical measures of muscle status suitable for routine care have not been evaluated for their association with long‐term survival and their relation with each other in a large cohort of KTR.

**Methods:**

Data of outpatient KTR ≥ 1 year post‐transplantation, included in the TransplantLines Biobank and Cohort Study (ClinicalTrials.gov Identifier: NCT03272841), were used. Muscle mass was determined as appendicular skeletal muscle mass indexed for height^2^ (ASMI) through bio‐electrical impedance analysis (BIA), and by 24‐h urinary creatinine excretion rate indexed for height^2^ (CERI). Muscle strength was determined by hand grip strength indexed for height^2^ (HGSI). Secondary analyses were performed using parameters not indexed for height^2^. Cox proportional hazards models were used to investigate the associations between muscle mass and muscle strength and all‐cause mortality, both in univariable and multivariable models with adjustment for potential confounders, including age, sex, body mass index (BMI), estimated glomerular filtration rate (eGFR) and proteinuria.

**Results:**

We included 741 KTR (62% male, age 55 ± 13 years, BMI 27.3 ± 4.6 kg/m^2^), of which 62 (8%) died during a median [interquartile range] follow‐up of 3.0 [2.3–5.7] years. Compared with patients who survived, patients who died had similar ASMI (7.0 ± 1.0 vs. 7.0 ± 1.0 kg/m^2^; *P* = 0.57), lower CERI (4.2 ± 1.1 vs. 3.5 ± 0.9 mmol/24 h/m^2^; *P* < 0.001) and lower HGSI (12.6 ± 3.3 vs. 10.4 ± 2.8 kg/m^2^; *P* < 0.001). We observed no association between ASMI and all‐cause mortality (HR 0.93 per SD increase; 95% confidence interval [CI] [0.72, 1.19]; *P* = 0.54), whereas CERI and HGSI were significantly associated with mortality, independent of potential confounders (HR 0.57 per SD increase; 95% CI [0.44, 0.81]; *P* = 0.002 and HR 0.47 per SD increase; 95% CI [0.33, 0.68]; *P* < 0.001, respectively), and associations of CERI and HGSI with mortality remained independent of each other (HR 0.68 per SD increase; 95% CI [0.47, 0.98]; *P* = 0.04 and HR 0.53 per SD increase; 95% CI [0.36, 0.76]; *P* = 0.001, respectively). Similar associations were found for unindexed parameters.

**Conclusions:**

Higher muscle mass assessed by creatinine excretion rate and higher muscle strength assessed by hand grip strength are complementary in their association with lower risk of all‐cause mortality in KTR. Muscle mass assessed by BIA is not associated with mortality. Routine assessment using both 24‐h urine samples and hand grip strength is recommended, to potentially target interdisciplinary interventions for KTR at risk for poor survival to improve muscle status.

## Introduction

For patients with end‐stage kidney disease (ESKD), kidney transplantation generally provides better quality of life and longer survival, compared with dialysis as a renal replacement therapy.^1^ Although short‐term outcomes after kidney transplantation have continued to improve over the last decades,^2^ long‐term survival of kidney transplant recipients (KTR) is still considerably lower than that of the general population.^3^ Previously identified, potentially modifiable risk factors for premature death in KTR include low physical activity and functioning^4^ and suboptimal nutritional status, reflected by weight gain, obesity^5^ and malnutrition.^6^

Low muscle mass goes hand‐in‐hand with physical inactivity and poor physical functioning.^7^, ^8^ Low muscle mass has also been shown to be an important characteristic of poor nutritional status in KTR.^9^ Previous reports showed a prevalence of low muscle mass of 19% to 50% in KTR, depending on the cut‐off value applied.^10^, ^11^ Using the diagnostic framework by the Global Leadership Initiative on Malnutrition (GLIM),^12^ we found low muscle mass to be a predominant phenotypic criterion of malnutrition, present in more than a quarter of outpatient KTR.^13^ Low muscle mass was also prevalent in KTR with a body mass index (BMI) in the overweight or obese range, stressing the importance of body composition assessment in this population.

Although muscle mass and muscle strength are related, they are not necessarily concordant.^14^ In a comparative study in male KTR and patients on peritoneal dialysis, no significant differences were found with regard to body composition assessed by dual‐energy X‐ray absorptiometry (DXA), while KTR had a significantly higher hand grip strength (HGS) compared with patients on peritoneal dialysis.^15^ In another study in 128 KTR ≥ 1 year post‐transplantation, low HGS was independently associated with a higher risk of hospitalization and all‐cause mortality, while low muscle mass according to assessment by bio‐electrical impedance analysis (BIA) was not.^16^ Importantly, in previous analyses, we found a strong association between muscle mass assessed by 24‐h urinary creatinine excretion rate (CER) and HGS.^17^ However, it remains unknown whether muscle mass and muscle strength are interchangeable or complementary to one another to identify patients at higher risk of all‐cause mortality. This information is necessary to optimally allow for pro‐active identification of KTR with suboptimal muscle status, who may benefit from interdisciplinary interventions to help prevent adverse outcomes. Therefore, in the current study, we aimed to evaluate the association between muscle mass and muscle strength and all‐cause mortality in KTR and in specific relation to one another. In primary analyses, muscle mass was assessed using appendicular skeletal muscle mass indexed to height^2^ (BIA) and the urinary creatinine excretion rate indexed to height^2^, and muscle strength was assessed using handgrip strength indexed to height^2^. In secondary analyses, similar analyses were performed using unindexed appendicular skeletal muscle mass (BIA), urinary creatinine excretion rate and unindexed handgrip strength.

## Materials and methods

### Study design and population

For the current prospective cohort study, data were extracted from the TransplantLines Biobank and Cohort Study of the University Medical Center Groningen (UMCG) (ClinicalTrials.gov Identifier: NCT03272841). All eligible transplant recipients who gave written informed consent were included in the TransplantLines cohort from June 2015 to the February 2021. The Medical Ethical Committee of the UMCG approved the TransplantLines study protocol (METc 2014/077), and all study procedures were performed in line with the principles of the Declaration of Helsinki. Further details on the design of the TransplantLines cohort have been described previously.^18^ For the current study, we included all adult KTR (≥18 years) enrolled in the TransplantLines study, with a functioning graft ≥1 year after transplantation and who had a scheduled study visit between June 2015 and February 2021 (*N* = 1144). Participants with missing data on the main variables regarding muscle mass and muscle strength (*N* = 374) were excluded, leaving 770 participants eligible for further analysis (participant flow chart is included in Figure S1). Based on an expected effect size of either 0.4^19^ or 2.4,^16^ 70 to 75 events were needed to attain sufficient statistical power (1‐β = 0.80) to detect a significant association, with a significance level of 0.05.

### Data collection

Anthropometric measures, that is, height, weight, waist and hip circumference, were performed using a wall‐secured stadiometer, a digital scale and a retractable measurement tape, respectively. Overweight was defined as BMI 25.0–29.9 kg/m^2^, and obesity was defined as BMI ≥ 30.0 kg/m^2^, according to the WHO classification. Body composition was assessed using a multi‐frequency BIA device (Quadscan 4000, Bodystat, Douglas, British Isles). Hand grip strength was measured using a hydraulic hand‐held dynamometer (Patterson Medical JAMAR 5030J1, Warrenville, Canada). Blood pressure was measured according to a standard clinical protocol using an automatic device (Philips Suresign VS2+, Andover, Massachusetts, USA).

Fasted blood samples and 24‐h urine samples were collected and analysed by using standard laboratory procedures, prior to the TransplantLines study visit. The serum creatinine‐based Chronic Kidney Disease Epidemiology Collaboration (CKD‐EPI) algorithm was used to calculate the estimated glomerular filtration rate (eGFR). Other laboratory measures included in this study were haemoglobin, high‐sensitivity C‐reactive protein (hs‐CRP), albumin, high‐density lipoprotein (HDL) cholesterol, low‐density lipoprotein (LDL) cholesterol, glucose and glycated haemoglobin (HbA1c) from blood samples. Creatinine clearance in mL/min was calculated by dividing 24‐h urinary creatinine excretion (mmol/24 h) by plasma creatinine (μmol/L), multiplying by 694 for conversion to mL/min. Proteinuria was defined as 24‐h urinary protein excretion ≥0.5 g/24 h.

Demographic variables and data on disease history, medication and transplant characteristics were extracted from the UMCG Renal Transplant Database.^18^ Follow‐up data on mortality were extracted from electronic hospital records and were updated using municipal records. Participants with unknown date of death (*N* = 1) were excluded from the analysis (Figure S1).

### Operationalization of main variables

#### Muscle mass by bio‐electrical impedance analysis

Outliers in BIA‐derived reactance and resistance, defined as a value of >1.5 IQR below Q1 or above Q3 (*N* = 18), were excluded for the analysis (Figure S1). Appendicular skeletal muscle mass (ASMM) was then calculated using BIA‐derived reactance and resistance, using the equation by Sergi et al.,^20^ in line with the GLIM criteria for malnutrition and the revised diagnostic criteria for sarcopenia (EWSGOP2).^7^, ^12^ Appendicular skeletal muscle mass index (ASMI), that is, ASMM in kg divided by height in meters squared, also in line with the GLIM and EWSGOP2 criteria, was used in the primary prospective analyses.

#### Muscle mass by 24‐h creatinine excretion rate

The 24‐h CER from urine collection samples was used as an alternative measure for muscle mass.^21^ Outliers in 24‐h CER, defined as a value of >1.5 IQR below Q1 or above Q3 (*N* = 6), were excluded for the analysis (Figure S1). The 24‐h urinary CER index (CERI), that is, 24‐h CER in mmol/24 h divided by height in meters squared, in line with the use of ASMI, was used in the primary prospective analyses.

#### Muscle strength

Hand grip strength was measured three times on both hands, and the maximum HGS value of all attempts was used for analysis. Outliers in HGS, defined as a value of >1.5 IQR below Q1 or above Q3 (*N* = 4), were excluded for the analysis (Figure S1). Hand grip strength index (HGSI), maximum HGS in kg divided by height in meters squared, again in line with use of ASMI, was used in the primary prospective analysis.

### Statistical analyses

Statistical analyses were performed using IBM SPSS Statistics for Windows, Version 23.0 and R version 4.0.3 (Vienna, Austria). Results were expressed as mean ± standard deviation (SD), median [interquartile range] or number (percentage) for normally distributed, skewed and categorical data, respectively. *χ* ^2^tests for categorical variables, independent samples *t*‐tests for normally distributed variables and Mann–Whitney *U*‐tests for skewed distributed or ordinal data were performed to determine differences in baseline characteristics between male and female participants. Pearson's correlation coefficient was determined to examine the association between the three main parameters of muscle status, that is, ASMI, CERI and HGSI, for male and female participants separately. The associations, stratified for sex, were visualized using scatterplots.

Cox proportional hazards models were used to investigate the associations of muscle mass and muscle strength with all‐cause mortality. Hazards ratios were computed per standard deviation increase. The proportional hazards assumption was verified visually with plots of the scaled Schoenfeld residuals and was not violated in any of the models. Potential effect‐modification by age or sex was explored by including product terms in the model. Adjustments were made for common potential confounders, including age and sex (Model 2) and additionally BMI (Model 3), and eGFR and proteinuria (Model 4). To avoid overfitting, additional models were created using additive adjustments to Model 4.^22^ Additive adjustments were made to Model 4 for inflammation (hs‐CRP and white blood cell count) (Model 5); glucose homeostasis (HbA1c, glucose and usage of antidiabetic drugs) (Model 6), transplantation related factors (pre‐emptive transplantation, living vs. deceased donor, usage of calcineurin inhibitors, usage of proliferation inhibitors and usage of mTOR inhibitors) (Model 7) and cardiovascular factors (blood pressure, HDL‐cholesterol and LDL‐cholesterol) (Model 8). To account for potential bias in prospective analyses that could result from the exclusion of participants with missing values in potential confounding variables (Table S1), multiple imputations were performed using fully conditional specification to obtain 10 imputed data sets.^23^ The algorithm was run for 30 iterations, and convergence of the Markov chains was evaluated with trace plots of the mean and variance. Analyses were performed in each of the data sets, and results were pooled using Rubin's rules.^23^ Primary analyses were performed using ASMI, CERI or HGSI. Secondary analyses were performed using unindexed ASMM, CER and HGS.

Finally, to investigate whether muscle mass by ASMI or CERI, or muscle strength by HGSI, are interchangeable or complementary to one another to identify patients at higher risk of all‐cause mortality, the prospective analyses were repeated according to the base model (Model 4), with additional adjustment for either ASMI, CERI or HGSI.

## Results

### Baseline characteristics

In total, 741 individual patients were included in the analyses (age 55 ± 13 years, 62% male, BMI 27.3 ± 4.6 kg/m^2^). Median [interquartile range] time after transplantation was 3.2 [1.0–9.4] years, 34% had a pre‐emptive transplantation, and 54% received a transplant from a living donor. Female participants more often had a pre‐emptive transplantation compared with men (39% vs. 31%; *P* = 0.03). eGFR was 51 ± 18 mL/min/1.73 m^2^, and 15% had proteinuria (median [IQR] proteinuria 1.3 [0.6–1.6] g/24 h). Prevalence of overweight and obesity according to BMI were 41% and 26%, respectively. Male participants more often were in the overweight range, while female participants more often were obese (*P* = 0.04). Baseline characteristics of the total study population, and for male and female participants separately, are shown in Table 1.

**Table 1:** Baseline characteristics of total population (*N* = 741)

	Total group	Males	Females	*P* value
*N* (%)	741 (100)	456 (62)	285 (38)	
Demographic variables				
Age (years)	55 ± 13	56 ± 16	55 ± 12	0.51
Ethnicity				0.29
Caucasian	693 (94)	426 (93)	267 (94)	
African	8 (1)	6 (1)	2 (1)	
Asian	11 (2)	5 (1)	6 (2)	
Other	11 (2)	9 (2)	2 (1)	
Unknown	18 (2)	10 (2)	8 (3)	
Medical history and medication				
Primary renal disease				0.91
Glomerulonephritis	169 (23)	109 (24)	60 (21)	
Interstitial nephritis	58 (8)	34 (8)	24 (8)	
Cystic kidney disease	139 (19)	80 (18)	59 (21)	
Other congenital/hereditary	32 (4)	21 (5)	11 (4)	
Renal vascular disease	64 (9)	40 (9)	24 (8)	
Diabetes mellitus	38 (5)	25 (6)	13 (5)	
Other multisystem disease	51 (7)	28 (6)	23 (8)	
Other	13 (2)	8 (2)	5 (2)	
Unknown	177 (24)	111 (24)	66 (23)	
Time after transplantation (years)	3.2 [1.0–9.4]	2.9 [1.0–9.5]	4.2 [1.0–9.2]	0.11
Type of transplantation				0.03
Pre‐emptive transplantation	255 (34)	143 (31)	112 (39)	
Dialysis prior transplantation	463 (63)	297 (65)	166 (58)	
Missing	23 (3)	16 (4)	7 (3)	
Type of donor				0.79
Living donor	395 (54)	240 (53)	155 (54)	
Postmortal donor	324 (44)	200 (44)	124 (44)	
Missing	22 (3)	16 (4)	6 (2)	
Immunosuppressive drugs				
Tacrolimus	506 (68)	324 (71)	182 (64)	0.04
Ciclosporin	111 (15)	56 (12)	55 (19)	0.01
Mycophenolic acid	560 (76)	353 (77)	207 (73)	0.14
Azathioprine	71 (10)	37 (8)	34 (12)	0.09
Prednisolone	720 (97)	447 (98)	273 (96)	0.07
Antidiabetic drugs				
Oral antidiabetic drugs	96 (13)	58 (13)	38 (13)	0.81
Insulin	83 (11)	48 (11)	35 (12)	0.46
Laboratory values and other clinical parameters^a^				
eGFR (mL/min/1.73m^2^)	51 ± 18	52 ± 17	50 ± 18	0.07
Creatinine clearance (mL/min)	69 ± 25	72 ± 25	65 ± 24	<0.001
Proteinuria	103 (15)	72 (17)	31 (12)	0.06
Haemoglobin (mmol/L)	8.3 ± 1.1	8.6 ± 1.1	7.9 ± 0.9	<0.001
CRP (mg/L)	2 [1–5]	2 [1–4]	2 [1–5]	0.56
White blood cell count (10^9^ cells/L)	7.7 ± 2.3	7.7 ± 2.1	7.7 ± 2.5	0.81
Albumin (g/L)	44 [42–46]	44 [42–46]	44 [42–45]	0.16
HDL‐cholesterol (mmol/L)	1.3 [1.1–1.7]	1.2 [1.0–1.5]	1.5 [1.2–1.9]	<0.001
LDL‐cholesterol (mmol/L)	2.8 [2.3–3.4]	2.8 [2.3–3.4]	2.9 [2.4–3.4]	0.29
Glucose (mmol/L)	5.5 [5.0–6.3]	5.6 [5.1–6.3]	5.3 [4.9–6.2]	0.01
HbA1c (mmol/mol)	40 [36–45]	40 [36–44]	40 [36–45]	0.94
Systolic blood pressure (mm Hg)	133 [124–144]	134 [125–144]	131 [122–144]	0.07
Anthropometric measurements				
Height (cm)	173 ± 9	178 ± 7	165 ± 7	<0.001
BMI (kg/m^2^)	27.3 ± 4.6	27.0 ± 4.1	27.7 ± 5.2	0.07
BMI category				0.04
Underweight (<18.5 kg/m^2^)	5 (1)	2 (0.4)	3 (1)	
Normal weight (18.5–24.9 kg/m^2^)	246 (33)	153 (34)	93 (33)	
Overweight (25.0–29.9 kg/m^2^)	301 (41)	199 (44)	102 (36)	
Obesity (≥30.0 kg/m^2^)	189 (26)	102 (22)	87 (31)	
Waist circumference (cm)	99 ± 14	102 ± 13	95 ± 14	<0.001
Muscle mass and muscle strength				
ASMM (kg)	21 ± 4	24 ± 3	18 ± 3	<0.001
ASMI (kg/m^2^)	7.0 ± 1.0	7.4 ± 0.9	6.4 ± 0.9	<0.001
CER (mmol/24 h)	11.8 [9.8–14.7]	13.4 [11.3–16.5]	10.1 [8.7–11.5]	<0.001
CERI (mmol/24 h/m^2^)	4.0 [3.4–4.8]	4.2 [3.6–5.1]	3.6 [3.1–4.2]	<0.001
HGS (kg)	38 ± 12	44 ± 10	28 ± 7	<0.001
HGSI (kg/m^2^)	12.4 ± 3.3	13.8 ± 3.0	10.1 ± 2.4	<0.001

*Note:* Data are presented as mean ± SD, number (%) or median [IQR].

Abbreviations: ASMI, appendicular skeletal muscle mass index; ASMM, appendicular skeletal muscle mass; BMI, body mass index; CER, 24‐h urinary creatinine excretion rate; CERI, 24‐h urinary creatinine excretion rate index; CRP, C‐reactive protein; eGFR, estimated glomerular filtration rate; HbA1c, glycated haemoglobin; HDL, high‐density lipoprotein; HGS, hand grip strength; HGSI, hand grip strength index; KTR, kidney transplant recipient(s); LDL, low‐density lipoprotein.

^a^ Due to missing data the number (*N*) available for analysis of proteinuria was *N* = 690; for haemoglobin, *N* = 737; for CRP, *N* = 738; for albumin, *N* = 739; for HDL‐ and LDL‐cholesterol, *N* = 735; for glucose, *N* = 713; for HbA1c, *N* = 730; and for systolic blood pressure, *N* = 730.

**Figure 1:**
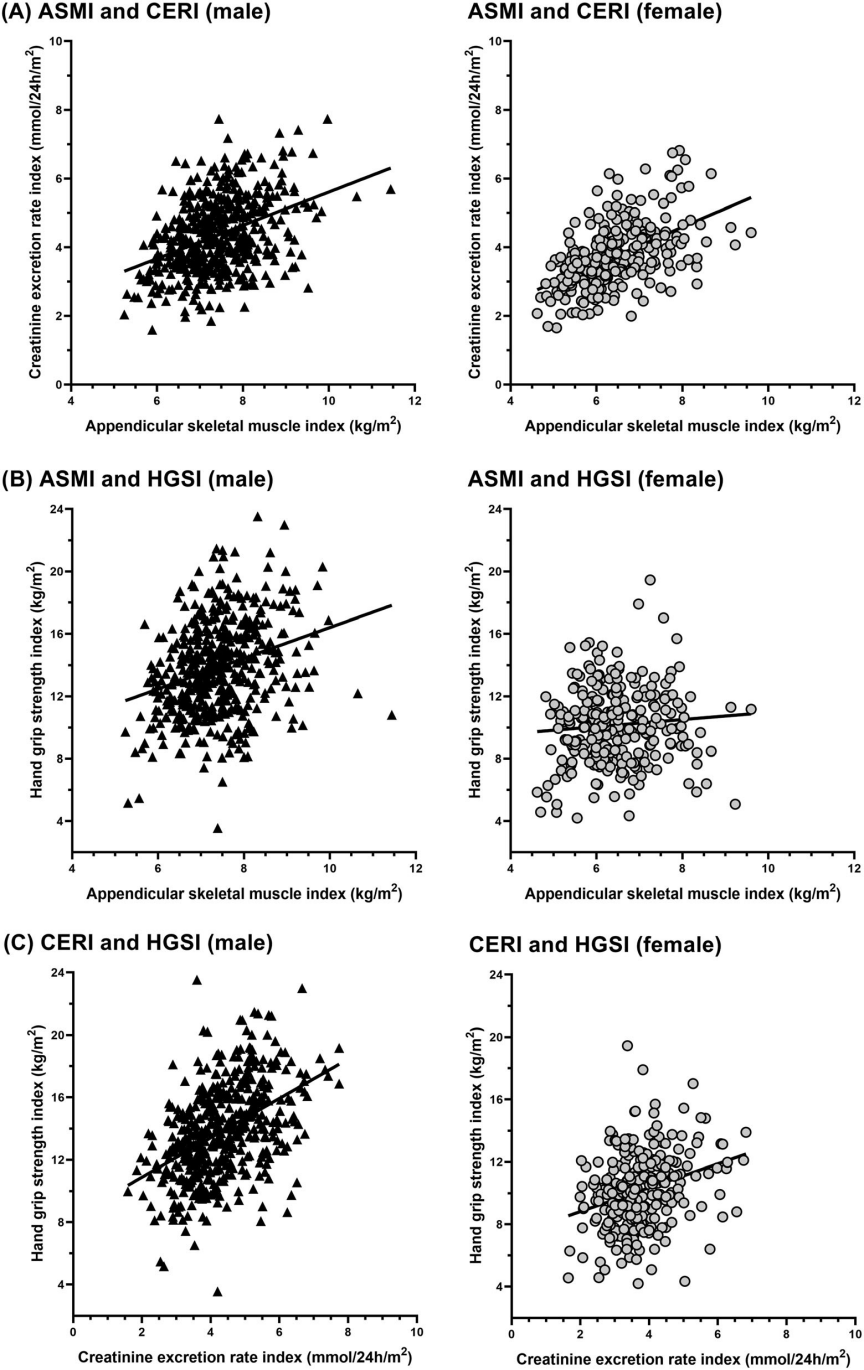
Correlations between main study variables, for males and females separately, (A) ASMI (kg/m^2^) and CERI (mmol/24 h/m^2^), (B) ASMI (kg/m^2^) and HGSI (kg/m^2^) and (C) CERI (mmol/24 h/m^2^) and HGSI (kg/m^2^). ASMI, appendicular skeletal muscle mass index; CERI, 24‐h urinary creatinine excretion rate index; HGSI, hand grip strength index.

As expected, male participants had a higher muscle mass based on ASMI/ASMM (7.4 ± 0.9 vs. 6.4 ± 0.9 kg/m^2^; *P* < 0.001 and 24 ± 3 vs. 18 ± 3 kg; *P* < 0.001, respectively) and CERI/CER (4.2 [3.6–5.1] vs. 10.1 [8.7–11.5] mmol/24 h/m^2^; *P* < 0.001 and 13.4 [11.3–16.5] vs. 10.1 [8.7–11.5] mmol/24 h; *P* < 0.001, respectively) and a higher muscle strength based on HGSI/HGS (13.8 ± 3.0 vs. 10.1 ± 2.4 kg/m^2^; *P* < 0.001 and 44 ± 10 vs. 28 ± 7 kg; *P* < 0.001, respectively), compared with female participants (Table 1). Correlations between the three main measures of muscle mass and strength indexed for height squared, stratified for sex, are visualized in Figure 1. ASMI was significantly correlated with CERI in males and females (males: *r* = 0.40; *P* < 0.001, females: *r* = 0.52; *P* < 0.001). ASMI was significantly correlated with HGSI in males (*r* = 0.29; *P* < 0.001), but not in females (*r* = 0.09; *P* = 0.16). CERI was significantly correlated with HGSI, in both males and females (males: *r* = 0.45; *P* < 0.001, females: *r* = 0.29; *P* < 0.001).

### Primary prospective analyses

A total of 62 out of 741 participants (8%) died during a follow‐up of 3.0 [2.3–5.7] years. Patients who died during follow‐up had similar ASMI to those who survived (7.0 ± 1.0 vs. 7.0 ± 1.0 kg/m^2^; *P* = 0.57), but lower CERI (3.5 ± 0.9 vs. 4.2 ± 1.1 mmol/24 h/m^2^; *P* < 0.001). In Cox‐regression analyses, ASMI was not significantly associated with risk of all‐cause mortality (HR 0.93 per SD increase; 95% CI [0.72, 1.19]; *P* = 0.46) (Model 1, Table 2). Further adjustment for other potential confounders did not materially change the association between ASMI and all‐cause mortality. For every standard deviation increase in CERI, the hazard ratio for all‐cause mortality was 0.52 (95% CI [0.38, 0.70]; *P* < 0.001) (Model 1, Table 2) and 0.57 (95% CI [0.40, 0.81]; *P* = 0.002), independent of age, sex, BMI, eGFR and proteinuria (Model 4, Table 2). Additional adjustment for inflammation markers (hs‐CRP and white blood cell count), glucose homeostasis, transplantation related factors or cardiovascular factors did not materially alter the association between CERI and all‐cause mortality (Models 5 to 8, Table 2). There was no effect‐modification according to age or sex (*P* > 0.05).

**Table 2:** Prospective analyses of muscle mass by ASMI, muscle mass by CERI and muscle strength by HGSI, with all‐cause mortality in 741 KTR

	ASMI (kg/m^2^)		CERI (mmol/24 h/m^2^)		HGSI (kg/m^2^)	
Model	HR [95% CI]	*P* value	HR [95% CI]	*P* value	HR [95% CI]	*P* value
1	0.92 [0.72, 1.19]	0.54	0.52 [0.38, 0.70]	<0.001	0.54 [0.41, 0.71]	<0.001
2	0.85 [0.62, 1.16]	0.30	0.59 [0.43, 0.82]	0.002	0.48 [0.35, 0.68]	<0.001
3	0.89 [0.54, 1.45]	0.63	0.58 [0.41, 0.83]	0.003	0.49 [0.35, 0.68]	<0.001
4	0.82 [0.50, 1.35]	0.43	0.57 [0.40, 0.81]	0.002	0.47 [0.33, 0.68]	<0.001
5	0.87 [0.53, 1.43]	0.58	0.59 [0.41, 0.85]	0.005	0.49 [0.34, 0.70]	<0.001
6	0.82 [0.50, 1.34]	0.42	0.58 [0.40, 0.83]	0.003	0.48 [0.33, 0.68]	<0.001
7	0.83 [0.51, 1.36]	0.45	0.59 [0.41, 0.85]	0.005	0.48 [0.33, 0.70]	<0.001
8	0.82 [0.50, 1.34]	0.42	0.58 [0.40, 0.82]	0.003	0.48 [0.33, 0.68]	<0.001

*Note:* All hazard ratios are presented per standard deviation increase of the variable of interest.

Model 1: Crude.

Model 2: Adjusted for age and sex.

Model 3: As Model 2, additionally adjusted for body mass index.

Model 4: As Model 3, additionally adjusted for proteinuria and eGFR.

Model 5: As Model 4, additionally adjusted for high sensitivity CRP and white blood cell count.

Model 6: As Model 4, additionally adjusted for glucose, HbA1c and usage of antidiabetic drugs.

Model 7: As Model 4, additionally adjusted for pre‐emptive transplantation, living vs deceased donor, usage of calcineurin inhibitors, proliferation inhibitors and mTOR inhibitors.

Model 8: As Model 4, additionally adjusted for systolic blood pressure, HDL‐cholesterol and LDL‐cholesterol.

Abbreviations: ASMI, appendicular skeletal muscle mass index; BIA, bio‐electrical impedance analysis; CERI, 24‐h urinary creatinine excretion rate index; CRP, C‐reactive protein; eGFR, estimated glomerular filtration rate; HbA1c, glycated haemoglobin; HDL, high‐density lipoprotein; HGSI, hand grip strength index; KTR, kidney transplant recipient(s); LDL, low‐density lipoprotein.

**Figure 2:**
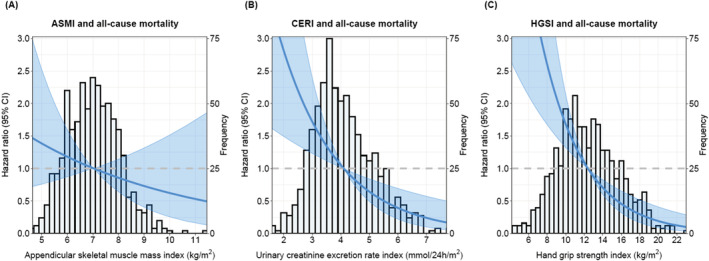
Graphical representation of the associations of ASMI, CERI and HGSI with all‐cause mortality. The lines show the adjusted hazard ratio (HR) and the shaded area corresponds to the 95% pointwise confidence interval (CI). The analyses were adjusted for age and sex. *P*_effect_ are 0.30, 0.002 and <0.001 for ASMI, CERI and HGSI, respectively.

Participants who died during follow‐up had lower HGSI (10.4 ± 2.8 vs. 12.6 ± 3.3 kg/m^2^; *P* < 0.001) than those who survived. For every standard deviation increase in HGSI, the hazard ratio for all‐cause mortality was 0.54 (95% CI [0.41, 0.71]; *P* < 0.001) (Model 1, Table 2) and 0.47 (95% CI [0.33, 0.68]; *P* < 0.001) (Model 4, Table 2) after subsequent adjustment for age, sex, BMI, eGFR and proteinuria. Further adjustment for potential confounders did not materially change the association (Models 5 to 8, Table 2). A graphical representation of the associations of ASMI, CERI and HGSI with all‐cause mortality is shown in Figure 2.

As expected, based on the results of the prospective analyses for the separate main variables, additional adjustment for ASMI did not materially alter the associations between CERI and HGSI with all‐cause mortality (base + ASMI, Table 3). When additionally adjusting for CERI in the base model, HGSI remained strongly associated with all‐cause mortality (HR 0.53 per SD increase; 95% CI [0.36, 0.76]; *P* = 0.001) (base + CERI, Table 3). And vice versa, when additionally adjusting for HGSI in the base model, CERI remained significantly associated with all‐cause mortality as well (HR 0.68 per SD increase; 95% CI [0.47, 0.98]; *P* = 0.04) (base + HGSI, Table 3).

**Table 3:** Prospective analyses of muscle mass by ASMI, muscle mass by CERI and muscle strength by HGSI, with all‐cause mortality in 741 KTR according to base model, with additional adjustment for either ASMI, CERI or HGSI

Model	ASMI (kg/m^2^)		CERI (mmol/24 h/m^2^)		HGSI (kg/m^2^)	
	HR [95% CI]	*P* value	HR [95% CI]	*P* value	HR [95% CI]	*P* value
Base	0.82 [0.50, 1.35]	0.43	0.57 [0.40, 0.81]	0.002	0.47 [0.33, 0.68]	<0.001
Base + ASMI	—	—	0.57 [0.40, 0.82]	0.003	0.47 [0.33, 0.67]	<0.001
Base + CERI	1.09 [0.68, 1.74]	0.72	—	—	0.53 [0.36, 0.76]	0.001
Base + HGSI	0.97 [0.61, 1.57]	0.91	0.68 [0.47, 0.98]	0.04	—	—

*Note:* All hazard ratios are presented per standard deviation increase of the variable of interest. Base model is adjusted for age and sex, body mass index, proteinuria and eGFR. Additive adjustments were made to the base model.

Abbreviations: ASMI, appendicular skeletal muscle mass index; BIA, bio‐electrical impedance analysis; CERI, 24‐h urinary creatinine excretion rate index; eGFR, estimated glomerular filtration rate; HGSI, hand grip strength index; KTR, kidney transplant recipient(s).

### Secondary prospective analyses

Prospective analyses with unindexed parameters for muscle mass and muscle strength, that is, ASMM, CER and HGS, yielded similar results to the primary prospective analyses with indexed parameters (Tables S2 and S3). ASMM was not significantly associated with risk of all‐cause mortality (HR 0.91 per SD increase; 95% CI [0.71, 1.17]; *P* = 0.46) (Model 1, Table S2). Further adjustment for other potential confounders did not materially change the association between ASMM and all‐cause mortality. For every standard deviation increase in CER, the hazard ratio for all‐cause mortality was 0.55 (95% CI [0.41, 0.74]; *P* < 0.001) (Model 1, Table S2) and 0.54 (95% CI [0.37, 0.80]; *P* = 0.002), independent of age, sex, BMI, eGFR and proteinuria (Model 4, Table S2). Additional adjustment for inflammation markers (hs‐CRP and white blood cell count), glucose homeostasis, transplantation related factors or cardiovascular factors did not materially alter the association between CER and all‐cause mortality (Models 5 to 8, Table S2). There was no effect‐modification according to age or sex (*P* > 0.05). For every standard deviation increase in HGS, the hazard ratio for all‐cause mortality was 0.59 (95% CI [0.44, 0.77]; *P* < 0.001) (Model 1, Table S2) and 0.46 (95% CI [0.30, 0.69]; *P* < 0.001) after subsequent adjustment for age, sex, BMI, eGFR and proteinuria (Model 4, Table S2). Further adjustment for potential confounders did not materially change the association.

The associations of CER and HGS with all‐cause mortality were both independent of each other (Table S3).

## Discussion

In this study, we aimed to investigate the association between measures of muscle mass and muscle strength with all‐cause mortality and in relation to each other, in outpatient KTR. We found that both low muscle mass by CERI and low muscle strength by HGSI were significantly associated with a higher all‐cause mortality, independent of other risk factors, whereas muscle mass determined by BIA‐derived ASMI was not. Importantly, muscle mass by CERI and muscle strength by HGSI also remained significantly associated with mortality, independent of each other. Our findings suggest assessment of muscle mass by urinary creatinine excretion and muscle strength by hand grip are of complementary use, to identify outpatient KTR with suboptimal muscle status at risk for poor survival. Routine assessment using both of these measures and pro‐active start of interdisciplinary interventions to improve muscle status in KTR are therefore recommended.

To our knowledge, this is the first study to evaluate muscle strength by HGSI/HGS and muscle mass by both BIA‐derived ASMI/ASMM and CERI/CER in the context of mortality risk in a large sample of KTR. Our findings are consistent with the results from a previous smaller study of 128 KTR ≥ 1 year post‐transplantation, in which low HGS, but not low muscle mass by BIA, was associated with a composite endpoint of mortality and hospitalization.^16^ Large‐scale prospective studies on the association between HGS and mortality in KTR are scarce, but HGS has previously shown to be a prognostic marker for mortality and health outcomes in the general population,^24^ the hospital population^25^ and in patients with chronic kidney disease (CKD).^26^ However, another study found that HGS was relatively preserved in patients with CKD and only found a crude association between decrease in HGS and higher risk of mortality, which lost statistical significance after adjustment for other risk factors.^27^

Prospective studies on the association between muscle mass and outcomes in KTR are also scarce, and use of different techniques for muscle mass assessment yielded mixed results. In the current study of adult outpatient KTR, we did not find an association between muscle mass as determined by BIA and all‐cause mortality. However, in a previous study of KTR aged 60 years or older, low muscle mass determined by CT at L3 vertebral level was significantly associated with longer hospitalization, higher rate of wound complications and higher rate of the combined endpoint of graft loss or death in male participants, but not in female participants.^28^ CT is considered to be one of the gold standard methods for muscle mass and quality assessment. However, for routine assessment and monitoring in clinical practice, muscle mass assessment by CT is not recommended, because of the radiation exposure, and CT scans are not usually available from routine care in patient groups other than cancer patients.^7^ In contrast, BIA is quick, cheap, portable and easily applicable, but its validity on the individual level is limited when assumptions on the stability of body fluids and other body compartments are not met, for example in obesity or in case of oedema or fluid shifts.^29^ Although our study included mostly stable outpatient KTR with relatively intact renal function (mean eGFR 51 ± 18 mL/min/1.73 m^2^), underlying renal disease, comorbidities and polypharmacy, including use of prednisolone, may have resulted in a less stable fluid status, influencing the validity of BIA. Also, because obesity was prevalent in more than a quarter of our study population, measurements of muscle mass by BIA might have been less valid. Overestimation of the fat‐free mass by BIA in obese participants may explain why muscle mass by BIA was not associated with all‐cause mortality in our study.

Previous studies comparing the accuracy of muscle mass assessment methods in KTR did show good accuracy to detect low muscle mass by BIA, compared with DXA and CT.^30^, ^31^ These studies, however, did not evaluate these methods in the context of patient outcomes, and their study populations differed from ours, for example, a longer time after transplantation in the Brazilian study^30^ and a lower BMI in the Japanese study.^31^ Also, in these previous studies, other BIA devices and other operationalization of muscle mass were used, while we used the equation by Sergi et al.^20^ to calculate ASSM, as recommended in the European sarcopenia consensus.^7^ However, the choice of equation probably had little influence, because our results did not materially change when we applied two other equations to calculate muscle mass (Table S4). The practical applicability of the BIA justifies further research on the predictive validity of BIA and the impact of different BIA devices and equations on its performance in KTR. Furthermore, other alternative, valid and easily applicable surrogate measures require further investigation, such as ultrasound^32^ or laboratory values/biomarkers, such as the creatinine/cystatin C ratio.^33^

Our results show that CERI/CER may be a useful alternative to BIA in assessing muscle mass of KTR. Although we found no association between muscle mass by BIA and all‐cause mortality, higher CERI/CER remained strongly associated with a lower risk of all‐cause mortality, independent of other potential confounders. Potential explanations for the discrepancy between these methods of muscle mass assessment in their association with mortality include the aforementioned methodological limitations of BIA in contrast to CER, which is not affected by other body compartments such as changes in fluid status and excess fat mass and may be more reflective of ‘functional’ muscle mass.^21^ Our results on the association between CERI/CER and all‐cause mortality are similar to the results from a previous large cohort of KTRs in our hospital, in which CER remained associated with both mortality and graft loss after adjustment for other characteristics.^19^ As a ‘multi‐use’ measure, 24‐h urine samples provide a relatively easy and low cost opportunity to assess muscle mass and other nutritional parameters at the same time, which is attractive for application in clinical practice. A limitation of the creatinine excretion rate may be the potential for underestimation in case of incomplete urinary collection. This may limit the generalization of its applicability in practice, although the experiences in our hospital and results from the current and previous studies show collection of 24‐h urine samples is quite feasible, given the infrastructure and organization allows for it. Another limitation of CER is that it requires patients to be in steady state, because several factors can influence CER if there is no steady state, that is, in case of active muscle wasting or recent trauma.

Importantly, in the current study, we found that muscle mass by CERI and muscle strength by HGSI are only moderately correlated, particularly in female KTR, and are complementary in their association with risk of all‐cause mortality. This finding suggests that muscle mass and muscle strength are related, but are separate domains. In the GLIM criteria for malnutrition (GLIM),^12^ low muscle mass is included as a phenotypic criterion for malnutrition which requires presence of an etiologic criterion for malnutrition diagnosis, whereas in the diagnostic framework for sarcopenia (EWGSOP2),^7^ low muscle strength is considered a key characteristic of sarcopenia, with presence of low muscle mass or quality to confirm the diagnosis. As we found both lower muscle strength and low muscle mass to be independent risk factors for all‐cause mortality, this points more in the direction of sarcopenia. However, only 9 (1%) of the KTR included in our study met the diagnostic criteria for sarcopenia, based on the proposed cut‐offs in the EWGSOP2‐criteria.^7^ This low number implies that either we are not looking at sarcopenia (or not fully) or maybe the proposed cut‐offs for the diagnosis of sarcopenia are not suitable for the KTR population. Our group recently reported that low muscle mass is a predominant criterion for the diagnosis of malnutrition,^13^ and lower protein intake is associated with severe fatigue, lower quality of life, and graft loss and mortality in KTR,^34^, ^35^ which suggests there is at least a nutritional component at play.

Together, our current findings and the findings from previous literature provide more insight in the complex interplay between body composition, functional status and nutritional intake in KTR. A useful paradigm to describe this complexity can be *anabolic competence*, which is defined as ‘a state which optimally supports protein synthesis and lean body mass, global aspects of muscle and organ function, and immune response’. This paradigm distinguishes three domains: nutrition, physical activity and the internal milieu. These three domains should be aligned to establish optimal body composition and physiologic function, and can be targeted by interdisciplinary, multimodal interventions.^36^ In KTR, these complex relations are likely further influenced by disease‐ and treatment‐related factors, such as previous dialysis treatment, and transplant‐specific factors, such as use of immunosuppressive drugs with catabolic effects. Future research is necessary to further unravel these complex relationships and characterize muscle and nutritional status, to work towards a feasible, tailored and interdisciplinary approach for improving outcomes in KTR.

Inflammation has been reported as an important factor in muscle wasting and mortality in the general population and in KTR.^37^ In the current study, adjustment for hs‐CRP and white blood cell count did not materially affect the association of HGSI and CERI with all‐cause mortality. Unfortunately, other inflammatory markers such as interleukin‐6 or TNF‐alpha were not available in our cohort, precluding further investigation using these markers of inflammation.

Our study has several implications for clinical practice. First, our findings support routine assessment of both muscle mass by CERI/CER and muscle strength by HGSI/HGS to identify KTR with suboptimal muscle status that are at risk of poor survival. Second, based on our findings, muscle mass assessment using BIA is not supported for this purpose in KTR. Lastly, the complex associations between muscle mass, muscle strength and outcomes, together with the concomitant high prevalence of obesity and malnutrition in KTR,^13^ stress the need for more integral and multimodal interventions to improve outcomes. Unimodal physical exercise interventions in KTR showed some beneficial effects on muscle performance and quality of life, but the effects on long‐term outcomes are not yet known.^38^ Nutrition and multimodal interventions in KTR are scarce, and the few available studies mostly focus on counteracting weight gain and reducing cardiovascular risk,^39^, ^40^ rather than the combination of cardiovascular risk management and improvement of muscle status and nutritional status from the perspective of anabolic competence. A potential future multimodal intervention may comprise a combination of, amongst others, but not limited to, nutritional counselling, supervised resistance training and psychosocial counselling and could be monitored using handgrip strength and creatinine excretion rate to assess efficacy. Nutritional counselling could be directed towards an adequate/higher intake of protein (including leucine content), optimal timing of meals, and sufficient intake or supplementation of omega‐3 fatty acids/fish oil and micronutrients, such as vitamin D.^35^, ^41^ The self‐reported importance of energy, fatigue, loss of strength and weight status for the health of transplant recipients further emphasizes the need for such multimodal interventions.^42^

The strength of the current study is that it is based on a large, well‐characterized cohort of KTR, the TransplantLines cohort. Therefore, multiple other risk factors for the primary endpoint all‐cause mortality could be accounted for in the prospective analyses. Due to the observational nature of the study design, no causal relationship between measures of muscle strength or muscle mass with mortality can be established. Therefore, we cannot distinguish whether the relationship of muscle mass and muscle strength with mortality is causal or associative. Although the current study is based on a large and mixed sample of outpatient KTR, all participants received care through a single university hospital in the Northern part of the Netherlands, which may limit the generalizability of our findings to other transplant care settings worldwide. Limitation of the current study are the lack of data on other aspects of muscle strength and function related to cardio fitness, such as a 6‐minwalking test, and detailed information on food intake, including longitudinal changes in those behaviours, as well as changes in muscle mass and muscle strength over time and muscle quality. Furthermore, we did not have data on hospitalization rates and were therefore unable to investigate the relationship of muscle mass and muscle strength with hospitalization. Future large‐scale studies are warranted to determine whether muscle mass and muscle strength are also associated with other relevant clinical endpoints including hospitalization or a composite endpoint of hospitalization and mortality.

In conclusion, both higher CERI/CER and higher HGSI/HGS are associated with a lower risk of all‐cause mortality in outpatient KTR ≥ 1 year post‐transplantation, independent of other potential prognostic factors. Furthermore, in a combined model of CERI and HGSI the association with mortality remained. The current study supports the use of both muscle mass assessment by urinary creatinine excretion rate and muscle strength assessment by hand grip to identify outpatient KTR with suboptimal muscle status that may benefit from interdisciplinary interventions to improve outcomes. Muscle mass assessment by BIA is not recommended to identify outpatient KTR at a higher risk of all‐cause mortality.

## Conflict of interest

The authors declare no conflicts of interest.

## Supporting information


**Figure S1.** *Participant flow chart. Abbreviations: BIA: bio‐electrical impedance analysis; CER: 24‐hour urinary creatinine excretion rate; HGS: hand grip strength.*
**Table S1.** *Overview of missing variables*
**Table S2**. *Prospective analyses of unindexed parameters of muscle mass and muscle strength, ASMM by BIA, CER and HGS, with all‐cause mortality in 741 KTR*
**Table S3.** *Prospective analyses of unindexed parameters of muscle mass and muscle strength, ASMM by BIA, CER and HGS, with all‐cause mortality in 741 KTR according to base model, with additional adjustment for either ASMM, CER or HGS*
**Table S4.** *Prospective analyses of alternative operationalizations and calculations of muscle mass using raw BIA data, with all‐cause mortality in 741 KTR*
