# Supplementary material for: Muscle mass, muscle strength and mortality in kidney transplant recipients: results of the TransplantLines Biobank and Cohort Study
Source: J Cachexia Sarcopenia Muscle. 2022 Oct 6;13(6):2932–43. doi: 10.1002/jcsm.13070 (PMC9745460; doi:10.1002/jcsm.13070)
Supplement: Supplementary file 1 — Figure S1. Participant flow chart. Abbreviations: BIA: bio‐electrical impedance analysis; CER: 24‐hour urinary creatinine excretion rate; HGS: hand grip strength. Table S1. Overview of missing variables Table S2. Prospective analyses of unindexed parameters of muscle mass and muscle strength, ASMM by BIA, CER and HGS, with all‐cause mortality in 741 KTR Table S3. Prospective analyses of unindexed parameters of muscle mass and muscle strength, ASMM by BIA, CER and HGS, with all‐cause mortality in 741 KTR according to base model, with additional adjustment for either ASMM, CER or HGS Table S4. Prospective analyses of alternative operationalizations and calculations of muscle mass using raw BIA data, with all‐cause mortality in 741 KTR [file JCSM-13-2932-s001.docx]

**Supplementary tables and figures**

**1144** participants, enrolled in the TransplantLines study, with a functioning graft ≥ 1 year after transplantation and a scheduled study visit between June 2015 and February 2021

**Excluded**:

- Missing data on main variables regarding muscle mass and muscle strength (*N=374*)

**770** participants potential eligible for analyses

**Excluded:**- Participants with unknown date of death (*N=1*)

- Participants with outliers (value >1.5IQR below Q1 or above Q3) in raw BIA data (*N=18*), CER (*N=6*) or HGS (*N=4*)

**741** participants included in the analyses

**Figure S1**. *Participant flow chart. Abbreviations: BIA: bio-electrical impedance analysis; CER: 24-hour urinary creatinine excretion rate; HGS: hand grip strength.*

| **Table S1.** *Overview of missing variables* | | |
| --- | --- | --- |
| **Variable** | **Number** | **Percentage (%)** |
| Urinary protein excretion | 51 | 6.9 |
| Systolic blood pressure | 11 | 1.5 |
| HDL cholesterol | 6 | 0.8 |
| LDL cholesterol | 6 | 0.8 |
| HbA1c | 11 | 1.5 |
| Glucose | 28 | 3.8 |
| Hs-CRP | 3 | 0.4 |
| Dialysis pre-emptive | 22 | 3.0 |
| Donor status | 22 | 3.0 |
| *Abbreviations: HbA1c: glycated haemoglobin; Hs-CRP: High sensitivity C-reactive protein* | | |

| **Table S2**. *Prospective analyses of unindexed parameters of muscle mass and muscle strength, ASMM by BIA, CER and HGS, with all-cause mortality in 741 KTR* | | | | | | |
| --- | --- | --- | --- | --- | --- | --- |
| **Model** | **ASMM (kg)** | | **CER (mmol/24h)** | | **HGS (kg)** | |
|  | HR [95% CI] | *P*-value | HR [95% CI] | *P*-value | HR [95% CI] | *P*-value |
| **1** | 0.91 [0.71 – 1.17] | 0.46 | 0.55 [0.41 – 0.74] | <0.001 | 0.59 [0.44 – 0.77] | <0.001 |
| **2** | 0.83 [0.57 – 1.21] | 0.33 | 0.56 [0.39 – 0.81] | 0.002 | 0.47 [0.32 – 0.70] | <0.001 |
| **3** | 0.89 [0.55 – 1.43] | 0.61 | 0.56 [0.38 – 0.82] | 0.003 | 0.48 [0.32 – 0.70] | <0.001 |
| **4** | 0.82 [0.50 – 1.34] | 0.42 | 0.54 [0.37 – 0.80] | 0.002 | 0.46 [0.30 – 0.69] | <0.001 |
| **5** | **0.86 [0.53 – 1.39]** | **0.52** | **0.56 [0.38 – 0.84]** | **0.004** | **0.47 [0.31 – 0.72]** | **<0.001** |
| **6** | 0.83 [0.51 – 1.35] | 0.44 | 0.55 [0.37 – 0.82] | 0.004 | 0.46 [0.31 – 0.69] | <0.001 |
| **7** | 0.82 [0.50 – 1.35] | 0.43 | 0.57 [0.38 – 0.84] | 0.005 | 0.47 [0.30 – 0.71] | <0.001 |
| **8** | 0.81 [0.50 – 1.32] | 0.40 | 0.55 [0.37 – 0.81] | 0.003 | 0.46 [0.31 – 0.69] | <0.001 |
| *All hazard ratios are presented per standard deviation increase of the variable of interest.*  *Model 1: Crude.*  *Model 2: Adjusted for age and sex.*  *Model 3: As model 2, additionally adjusted for BMI.*  *Model 4: As model 3, additionally adjusted for proteinuria and eGFR.*  *Model 5: As model 4, additionally adjusted for high sensitivity CRP* ***and white blood cell count****.*  *Model 6: As model 4, additionally adjusted for glucose, HbA1c and usage of antidiabetic drugs.*  *Model 7: As model 4, additionally adjusted for pre-emptive transplantation, living vs deceased donor, usage of calcineurin inhibitors, proliferation inhibitors and mTOR inhibitors.*  *Model 8: As model 4, additionally adjusted for systolic blood pressure, HDL-cholesterol and LDL-cholesterol.*  *Abbreviations: ASMM: appendicular skeletal muscle mass ; BIA: bio-electrical impedance analysis; CER: 24-hour urinary creatinine excretion rate;*  *CRP: C-reactive protein; eGFR: estimated glomerular filtration rate; HbA1c: glycated haemoglobin; HDL: high density lipoprotein; HGS: hand grip strength; KTR: kidney transplant recipient(s);*  *LDL: low density lipoprotein.* | | | | | | |

| **Table S3.** *Prospective analyses of unindexed parameters of muscle mass and muscle strength, ASMM by BIA, CER and HGS, with all-cause mortality in 741 KTR according to base model, with additional adjustment for either ASMM, CER or HGS* | | | | | | |
| --- | --- | --- | --- | --- | --- | --- |
| **Model** | **ASMM (kg)** | | **CER (mmol/24h)** | | **HGS (kg)** | |
|  | HR [95% CI] | *P*-value | HR [95% CI] | *P*-value | HR [95% CI] | *P*-value |
| **Base** | 0.85 [0.52 – 1.37] | 0.49 | 0.56 [0.37 – 0.82] | 0.004 | 0.47 [0.31 – 0.71] | <0.001 |
| **Base + ASMM** | - | - | 0.54 [0.36 – 0.80] | 0.003 | 0.43 [0.28 – 0.66] | <0.001 |
| **Base + CER** | 1.04 [0.64 – 1.71] | 0.87 | - | - | 0.53 [0.34 – 0.82] | <0.001 |
| **Base + HGS** | 1.23 [0.75 – 2.04] | 0.40 | 0.67 [0.44 – 1.01] | 0.05 | - | - |
| *All hazard ratios are presented per standard deviation increase of the variable of interest.*  *Base model is adjusted for age and sex, BMI, proteinuria and eGFR. Additive adjustments were made to the base model.*  *Abbreviations: ASMM: appendicular skeletal muscle mass; BIA: bio-electrical impedance analysis; CER: 24-hour urinary creatinine excretion rate; HGS: hand grip strength; KTR: kidney transplant recipient(s).* | | | | | | |

| **Table S4.** *Prospective analyses of alternative operationalizations and calculations of muscle mass using raw BIA data, with all-cause mortality in 741 KTR* | | | | | | | | |
| --- | --- | --- | --- | --- | --- | --- | --- | --- |
| **Model** | **ASMM Kyle (kg)** | | **ASMI Kyle (kg/m^2^)** | | **FFM Kyle (kg)** | | **FFMI Kyle (kg/m^2^)** | |
|  | HR [95% CI] | *P*-value | HR [95% CI] | *P*-value | HR [95% CI] | *P*-value | HR [95% CI] | *P*-value |
| **1** | 0.92 [0.72 – 1.19] | 0.52 | 0.94 [0.73 – 1.21] | 0.63 | 0.93 [0.72 – 1.19] | 0.56 | 0.95 [0.74 – 1.23] | 0.70 |
| **2** | 0.85 [0.58 – 1.26] | 0.42 | 0.88 [0.63 – 1.21] | 0.41 | 0.84 [0.57 – 1.24] | 0.37 | 0.86 [0.63 – 1.18] | 0.35 |
| **3** | 0.93 [0.58 – 1.50] | 0.76 | 0.96 [0.59 – 1.54] | 0.86 | 0.90 [0.55 – 1.49] | 0.69 | 0.92 [0.55 – 1.53] | 0.74 |
| **4** | 0.86 [0.52 – 1.41] | 0.54 | 0.88 [0.54 – 1.43] | 0.61 | 0.83 [0.50 – 1.39] | 0.47 | 0.85 [0.51 – 1.42] | 0.53 |
| **5** | **0.90 [0.55 – 1.46]** | **0.66** | **0.94 [0.58 – 1.52]** | **0.79** | **0.87 [0.53 – 1.44]** | **0.59** | **0.90 [0.54 – 1.51]** | **0.69** |
| **6** | 0.86 [0.53 – 1.41] | 0.54 | 0.88 [0.54 – 1.42] | 0.59 | 0.84 [0.50 – 1.39] | 0.48 | 0.84 [0.51 – 1.40] | 0.50 |
| **7** | 0.87 [0.53 – 1.43] | 0.57 | 0.89 [0.55 – 1.45] | 0.63 | 0.84 [05.0 – 1.42] | 0.51 | 0.86 [0.51 – 1.44] | 0.56 |
| **8** | 0.85 [0.52 – 1.38] | 0.50 | 0.88 [0.54 – 1.41] | 0.58 | 0.82 [0.50 – 1.37] | 0.45 | 0.85 [0.51 – 1.40] | 0.51 |
| *All hazard ratios are presented per standard deviation increase of the variable of interest.*  *Model 1: Crude.*  *Model 2: Adjusted for age and sex.*  *Model 3: As model 2, additionally adjusted for BMI.*  *Model 4: As model 3, additionally adjusted for proteinuria and eGFR.*  *Model 5: As model 4, additionally adjusted for high sensitivity CRP* ***and white blood cell count****.*  *Model 6: As model 4, additionally adjusted for glucose, HbA1c and usage of antidiabetic drugs.*  *Model 7: As model 4, additionally adjusted for pre-emptive transplantation, living vs deceased donor, usage of calcineurin inhibitors, proliferation inhibitors and mTOR inhibitors.*  *Model 8: As model 4, additionally adjusted for systolic blood pressure, HDL-cholesterol and LDL-cholesterol.*  *Abbreviations: ASMM: appendicular skeletal muscle mass ; BIA: bio-electrical impedance analysis; CER: 24-hour urinary creatinine excretion rate;*  *CRP: C-reactive protein; eGFR: estimated glomerular filtration rate; HbA1c: glycated hemoglobin; HDL: high density lipoprotein; HGS: hand grip strength; KTR: kidney transplant recipient(s);*  *LDL: low density lipoprotein.* | | | | | | | | |
